# From Pollen to Pathogen Defense: How Pollen Chemical Quality Impacts Deformed Wing Virus Infection and Survival in Honey Bees

**DOI:** 10.3390/v18070695

**Published:** 2026-06-24

**Authors:** Richard García Domínguez, María D. López-Belchí, Nolberto Arismendi, Marisol Vargas

**Affiliations:** 1 Laboratories of Virology and Bee Pathology, Faculty of Agronomy, Universidad de Concepcion, Chillán 3780000, Chile; rgarciad@udec.cl; 2 Department of Plant Production, Faculty of Agronomy, University of Concepción, Chillán 3780000, Chile; mlopezb@udec.cl; 3 Austral Biotech Research Centre, Faculty of Science, University of Santo Tomás, Valdivia 5090000, Chile; narismendi@santotomas.cl

**Keywords:** nutrition, *Apis mellifera*, DWV, phytochemicals, innate immunity, gut microbiota

## Abstract

Pollen constitutes the primary source of proteins, amino acids, lipids, sterols, vitamins, and minerals for honey bees. However, not all pollen types provide the same resources or have the same biological value. Its chemical composition changes according to botanical origin, geographic location, and environmental conditions. This variability can influence metabolism, the immune system, oxidative balance, and the ability to resist or tolerate infections. This article examines the available evidence on the relationship between pollen chemical quality and the dynamics of Deformed Wing Virus (DWV) infection in *Apis mellifera*. The analysis is approached from molecular, physiological, ecological, and seasonal perspectives. Current findings suggest that more diverse and higher-quality pollen diets are generally associated with greater colony survival and improved health status, although their effects on viral load are more heterogeneous and context-dependent. In some studies, pollen intake is linked to a reduction in DWV, whereas in others viral loads remain stable or even increase despite improvements in survival, physiological condition, or colony performance. These differences suggest that pollen may act not only by enhancing resistance to the virus but also by increasing tolerance to infection-associated damage. The potential role of pollen bioactive compounds, particularly flavonoids and phenolic acids, is also discussed. Nevertheless, evidence of direct antiviral action of these compounds in bees remains limited, as many proposed mechanisms derive from other organisms. This synthesis provides an integrative perspective on pollen nutrition and its relevance for colony resilience against viral infections.

## 1. Introduction

The health of honey bees (*Apis mellifera* L.) is essential for the functioning of terrestrial ecosystems and global agricultural production because of their central role in the pollination of both crops and wild plants. Pollination by animals underpins the reproduction of nearly 90% of wild flowering plants, contributes to more than 75% of global food crop types, and supports an estimated US$235–577 billion in annual crop production worldwide [1] (Priyadarshana et al., 2024). However, over the past decades, a sustained decline in colony hea lth has been documented across different regions of the world, largely driven by the interaction of multiple stressors, including pathogens, agricultural intensification, pesticide exposure, and reduced floral diversity [2].

Among the biological factors compromising colony stability, viral infections are particularly important because of their high prevalence and cumulative sublethal effects. DWV has emerged as one of the most important viral pathogens of *A. mellifera* due to its widespread distribution, persistence, and close association with the mite *Varroa destructor*, which promotes its transmission and amplification within colonies [3]. DWV infection has been associated with alterations in development, physiology, behavior, and lifespan, thereby contributing to the progressive weakening of colonies [4].

In honey bees, defense against pathogens relies primarily on innate immune mechanisms. At the colony level, these defenses are complemented by forms of social immunity, such as grooming behavior and the removal of compromised individuals, which help limit pathogen transmission [5,6]. Nevertheless, the effectiveness of these defenses depends strongly on the physiological condition of bees and on the nutritional context in which infection occurs [7]. Within this framework, nutrition has been proposed to influence not only the activation of immune responses but also the host capacity to tolerate damage associated with persistent viral infections [8].

In this context, the nutrition of honey bees encompasses the acquisition and utilization of essential macro- and micronutrients that sustain individual growth, development, and vital functions. Carbohydrates, obtained from nectar, represent the primary energy source and contribute to oxidative balance and antimicrobial defense [9]. Pollen, on the other hand, provides proteins, amino acids, lipids, sterols, vitamins, minerals, and bioactive compounds that support tissue development and immune competence, although its nutritional quality varies according to botanical origin and environmental conditions [10,11].

Despite growing interest in the links between nutrition and honey bee health, the specific role of pollen chemical quality in shaping DWV infection outcomes remains insufficiently integrated. Available evidence is dispersed across studies focused on nutrition, immune function, oxidative stress, gut microbiota, and seasonal ecology, and is often difficult to compare because of differences in experimental systems, life stages, and response variables. In this systematic review, the available evidence linking pollen chemical quality to DWV infection dynamics and survival in *A. mellifera* is examined across molecular, physiological, ecological, and seasonal scales. Attention is given to the role of bioactive pollen compounds, especially flavonoids and phenolic acids, while explicitly distinguishing evidence derived from honey bee studies from mechanistic hypotheses proposed in other viral systems. This review aims to integrate these fragmented lines of evidence, identify the main knowledge gaps, and provide a critical framework for understanding how pollen chemical quality may shape DWV outcomes and honey bee survival under different biological and ecological contexts.

## 2. Methodology

This review was conducted as a structured narrative synthesis given the substantial heterogeneity in viral inoculation methods, host age, diet composition, and outcome metrics in the literature. Comprehensive research was performed in Web of Science, Scopus, PubMed, Google Scholar, and journal databases using combinations of terms related to bee nutrition, DWV infection, immune gene expression, pollen chemistry, phytochemicals, and microbiota. The primary search window covered studies published between 2000 and 2026, with older references retained only when they provided foundational information on honey bee immunity or viral biology. Priority was given to peer-reviewed experimental studies evaluating pollen, pollen substitutes, bee bread, floral phytochemicals, or defined dietary compounds in relation to DWV or other honey bee viruses. Studies were grouped by experimental scale, dietary treatment, pathogen context, and response variables, allowing mechanistic and ecological evidence to be integrated qualitatively and comparatively.

## 3. Deformed Wing Virus (DWV) in *A. mellifera*: Biology, Transmission, and Pathological Effects

DWV is one of the most widely distributed viral pathogens in *A. mellifera* and is currently recognized as one of the central components within the set of factors underlying colony weakening and collapse at a global scale [12]. Its high prevalence, even in colonies that do not show clinical signs, reflects its remarkable ability to establish persistent and subclinical infections, which can be maintained for prolonged periods within bee populations [13]. This characteristic contributes to the persistence of DWV within honey bee populations and helps explain why its impact is often expressed progressively and cumulatively, rather than as isolated events of massive mortality [14]. DWV belongs to the family *Iflaviridae* and corresponds to a non-enveloped, positive-sense single-stranded RNA virus, whose genomic organization follows the characteristic pattern of picorna-like viruses [15]. Its genome is translated as a single polyprotein that is subsequently processed to give rise to structural and non-structural proteins, including an RNA-dependent RNA polymerase, a helicase, and a 3C-like protease, all of which are indispensable for viral replication and virion assembly [16]. In recent years, advances based on reverse genetics have made it possible to deepen the understanding of the replicative biology of DWV; however, relevant gaps remain regarding its cellular tropism and the host factors that modulate the efficiency of viral replication [17]. At the population level, DWV should not be understood as a homogeneous entity, but rather as a complex of closely related genetic variants. To date, at least four main variants have been described, DWV-A, DWV-B, DWV-C and DWV-D, in addition to multiple recombinant forms whose relative frequencies vary as a function of space and time [18,19].

DWV transmission can occur through multiple routes, including vertical transmission from the queen to the offspring through drone semen, horizontal transmission between individuals by trophallaxis, and contact with infected pupae [20,21]. Nevertheless, the most consequential change in the epidemiology of this virus has been its association with vector-mediated transmission by *V. destructor*, a process that significantly increases transmission efficiency and promotes abrupt increases in viral load. In addition, this route is associated with a greater probability of clinical disease manifestation [12,22]. The association between DWV and *V. destructor* has therefore redefined not only the intensity of infection, but also its pathological and epidemiological consequences within colonies [23]. In this sense, it has been described that DWV replication can occur in diverse bee tissues, and that tissue tropism constitutes a key component for understanding the breadth of the physiological effects associated with infection [24]. When high viral loads are reached during pupal development, particularly in the presence of *V. destructor* parasitism, evident clinical manifestations may appear, including the characteristic deformed wing phenotype [25]. However, in most adult bees, DWV persists as a covert infection, with sublethal consequences that include reduced longevity, lower survival, and impaired foraging performance [26]. Therefore, the biological importance of DWV lies not only in its capacity to generate visible clinical signs, but also in its ability to silently and persistently compromise colony physiology and functioning.

## 4. Immune System of Honey Bees and Its Modulation by Diet Quality

Since the pathological manifestation of DWV depends not only on viral load but also on the host’s ability to resist and tolerate infection, it is essential to consider the organization of the immune system in *A. mellifera* and the nutritional factors that modulate its function. Honey bees rely on an innate immune system to combat pathogens, as they lack antibody-based adaptive immunity. This system is composed of humoral and cellular mechanisms highly conserved in insects, allowing bees to recognize, limit, and tolerate infections caused by bacteria, fungi, and viruses [5]. The effectiveness of these responses is not constant but is closely linked to the physiological state of the individual, which depends largely on the availability and quality of nutritional resources [27,28]. However, some studies have shown that adequate pollen nutrition does not necessarily reduce DWV titers and that, under certain conditions, viral loads may remain unchanged or even increase [29,30]. From the perspective of host defense theory, these contrasting outcomes can be interpreted within the resistance–tolerance framework [31]. Resistance mechanisms reduce pathogen burden, whereas tolerance mechanisms reduce the physiological consequences of infection without necessarily decreasing viral load. This distinction is particularly relevant for DWV because improved nutrition may enhance bee survival and colony performance even when viral titers remain unchanged [32].

Pathogen recognition in *A. mellifera* occurs through pattern recognition receptors (PRRs), which detect conserved molecular structures of microorganisms and activate intracellular signaling cascades [33]. Among the main immune pathways described in bees are Toll, Imd, JAK/STAT, and JNK, which regulate the expression of immune genes and the production of antimicrobial peptides (AMPs) such as defensin, abaecin, apidaecin, and hymenoptaecin [5,34].

Against RNA viruses such as DWV, the primary antiviral defense in bees is RNA interference (RNAi), a mechanism highly conserved in insects [35]. During viral replication, double-stranded RNA (dsRNA) intermediates are generated, recognized by the enzyme Dicer-2 and processed into small interfering RNAs (siRNAs) [36]. These siRNAs are incorporated into the RISC complex, where Argonaute-2 directs the specific degradation of viral RNA, thereby reducing viral replication [37]. The activity of the RNAi pathway is energetically demanding, and its efficiency is thus directly sensitive to nutritional status [38]. Together with the production of antimicrobial peptides and the activation of immune signaling pathways, RNAi is primarily associated with resistance because it contributes directly to limiting viral replication. A non-specific antiviral response induced by dsRNA has also been described in *A. mellifera*, capable of activating immune genes without exact sequence matching [39].

Along with humoral responses, bees possess cellular immunity mechanisms mediated by hemocytes, which participate in phagocytosis, encapsulation, and melanization [40]. Melanization, regulated by the enzyme phenoloxidase, generates reactive oxygen species as by-products, implying a risk of collateral tissue damage if not properly controlled [41]. This balance between immune activation and damage control is particularly relevant during persistent infections such as DWV and represents an interface where pollen-derived antioxidants may play a meaningful physiological role. Such processes are more closely aligned with tolerance mechanisms, as they may reduce infection-associated damage and improve host survival even when viral loads remain unchanged [8].

The activation and maintenance of immune responses represent a significant energetic cost [38]. In this context, poor or low-diversity pollen is associated with reduced immune capacity and increased susceptibility to viral infections, whereas access to high-quality pollen improves general physiological condition and tolerance to pathogenic stress [42]. The effectiveness of these immune pathways, both humoral and cellular, depends not only on their genetic integrity, but on the availability of the energetic and molecular substrates that pollen provides. Dietary quality thus emerges as a transversal determinant of immune function in *A. mellifera*, ultimately shaping the margin of resistance and tolerance available against DWV infection.

## 5. Pollen Chemical Quality as a Determinant of Defense Against Deformed Wing Virus (DWV) in *A. mellifera*

In *A. mellifera*, pollen constitutes the primary dietary source of proteins, lipids, sterols, vitamins, minerals, and a wide range of bioactive compounds [43]. These elements provide the chemical substrates necessary to sustain the vital processes related to the health and defense of the host organism [44]. Unlike nectar, whose role is primarily energetic, pollen supplies the essential compounds that become especially critical under pathogen pressure [45]. Pollen quality is shaped by multiple factors, including protein content, amino acid composition, lipid concentration, antioxidant capacity, sterol availability, and the balance among macronutrients, particularly protein-to-lipid (P:L) ratios. Honey bees can selectively forage on pollen resources that better match their nutritional requirements, and access to diverse floral communities may facilitate nutritional balancing through complementary pollen sources [46]. The magnitude of this nutritional dependence becomes evident when considering the scale at which colonies consume pollen. A colony can consume between 15 and 55 kg annually, with individual adult bee daily consumption ranging from 3.4 to 5.4 mg depending on age [11]. The period of greatest pollen demand corresponds to the phases of active brood rearing and population growth. To rear a single worker larva, between 124 and 187.5 mg of pollen are required, providing between 25 and 37.5 mg of protein depending on the available protein content [47]. This demand falls primarily on nurse bees, which during their first 10 days of age consume large quantities of pollen to drive the development and secretory activity of their hypopharyngeal glands [48]. These glands produce the main protein fraction of royal jelly, distributed via trophallaxis to larvae and the queen. Therefore, the nutritional status of nurse bees is not merely an individual matter, but a determining factor of population dynamics.

These figures reflect not only the energetic investment that pollen represents for the colony, but also its indispensable role in sustaining all major physiological functions [49]. However, pollen composition is far from uniform. Protein levels ranging from 2.5% to 61% have been reported in pollen collected by honey bees [50]. This variability in quantity and quality can represent a challenge for the nutritional homeostasis of the colony [51]. Nutritional deficits are directly reflected in lifespan, disease susceptibility, and body weight. In this regard, Di Pasquale et al. [28] reported substantial variation in the nutritional composition of pollen from different botanical origins. *Rubus* pollen contained 22% protein, 19.98 g amino acids per 100 g of pollen, and an antioxidant capacity of 475 µmol Trolox equivalents g^−1^ pollen, whereas *Cistus* pollen contained 12% protein, 11.9 g amino acids per 100 g of pollen, and an antioxidant capacity of 103 µmol Trolox equivalents g^−1^ pollen. Correspondingly, bees fed *Rubus* pollen developed larger hypopharyngeal glands and exhibited higher vitellogenin and transferrin expression than bees fed *Cistus* pollen. Similarly, Branchiccela et al. [32] found that nutritional stress associated with a diet based primarily on *E. grandis* pollen compromised colony strength, observing a reduction in brood and adult bee populations. While supplemented colonies showed higher levels of RNA virus infection, these were generally low and without a clear negative impact. The authors suggest that improved nutrition may favor a more active physiological machinery that promotes viral replication, while also enhancing the colonies’ ability to cope with infection. Furthermore, lower *Nosema* infection in supplemented colonies may have reduced competition for nutritional resources, potentially contributing to greater DWV replication.

In this context, a previous study from our research group [52] evaluated the effect of diets based on pollen of different botanical compositions on DWV-A viral load, survival, and immune gene expression in experimentally inoculated bees. The results showed that native *E. cordifolia* pollen drastically reduced viral load from 1.0 × 10^13^ to 1.0 × 10^5^ copies per bee, along with a survival rate of 91%. Furthermore, pollen diets modulated the expression of immune-related genes, reducing *Cactus* expression while upregulating *Dorsal*, *Relish*, and *Dicer-like*. At the colony level, DeGrandi-Hoffman et al. [53] demonstrated that colonies supplemented with pollen developed larger populations and survived longer than unsupplemented ones, although *V. destructor* and DWV levels increased similarly in both groups throughout the season. This suggests that the primary benefit of pollen does not lie in directly reducing pathogen load, but in strengthening colony resilience against infection. As shown in Table 1, these findings indicate that pollen quality influences susceptibility to DWV across multiple levels of biological organization, from nurse bee physiology and gland development to immune gene expression, brood quality, population dynamics, and seasonal colony resilience. Consequently, pollen should be understood not only as a nutritional resource but also as a key modulator of host–pathogen interactions in *A. mellifera*, with its quality and diversity shaping the colony’s physiological capacity to tolerate viral infections such as DWV. Nevertheless, stored pollen (bee bread) may also contribute to viral transmission within the colony. Bee bread can harbor infective DWV particles and may facilitate viral persistence, particularly in colonies with high DWV prevalence, a condition frequently associated with *V. destructor* infestation [54].

## 6. Pollen Phytochemicals as Context-Dependent Modulators of DWV Outcomes in *A. mellifera*

Pollen is not only a source of essential nutrients for *A. mellifera*, but also a chemically complex matrix of secondary metabolites with potentially important effects on host physiology and pathogen responses. Among the most relevant compounds are polyphenols, terpenoids, phytosterols, alkaloids, and other aromatic metabolites, whose abundance and composition vary markedly with botanical origin, floral diversity, and environmental conditions [59,60]. Phenolic compounds are particularly prominent in this context because they are frequently detected in pollen and have been associated with antioxidant activity and other biological properties that may influence bee health [51].

In honey bees, the relevance of pollen-derived phytochemicals extends beyond their mere presence in floral resources [38]. Current evidence suggests that these compounds can modulate immune function, oxidative balance, and physiological resilience, thereby influencing the host context in which viral infection develops. At the molecular level, phytochemicals derived from nectar and pollen have been associated with changes in the expression of antiviral effectors and immune-related signaling pathways, including the Toll and Imd pathways [61,62]. However, the magnitude and biological relevance of these responses appear to depend on both the specific compound involved and the physiological status of the bees.

Some of the strongest evidence in bees comes from experimental studies demonstrating that dietary phytochemicals can modulate immune responses, alter antimicrobial peptide expression, improve survival, and influence DWV-related outcomes. Palmer-Young et al. [63] reported that phytochemicals naturally present in nectar and pollen stimulated antimicrobial peptide expression in adult bees and reduced DWV levels in young bees under experimental conditions. Likewise, Lu et al. [64] showed that caffeine increased the expression of immune-related genes and reduced DWV copy number in *A. mellifera*. More broadly, dietary phytochemicals have also been linked to increased longevity and improved tolerance to pathogens, reinforcing the view that floral chemistry may shape infection outcomes not only through effects on pathogen load, but also through enhanced host resilience [65].

Beyond immune modulation, pollen-derived phytochemicals may influence host–pathogen interactions through several non-exclusive mechanisms. They may contribute to antioxidant protection, help maintain redox homeostasis during infection, and support physiological functions that are otherwise compromised under viral challenge [66]. They may also interfere more directly with viral processes, although evidence for this possibility remains much stronger in non-bee systems than in honey bees themselves [37,67]. Because direct mechanistic evidence linking pollen-derived phytochemicals to DWV infection outcomes in honey bees is still limited, much of the current functional interpretation relies on studies from other RNA virus models, where polyphenols have been shown to act at different stages of the viral cycle.

Nevertheless, structural studies of DWV have provided insights into potential viral targets that could be susceptible to chemical interference. The high-resolution structure of the DWV virion revealed a surface-exposed P domain containing conserved putative catalytic residues that may participate in receptor binding and viral entry, as well as conserved RNA-binding sites involved in virion stability and assembly [68]. Although direct evidence of phytochemical interactions with these targets is currently lacking, these structural insights provide a useful mechanistic framework for future studies investigating how pollen-derived compounds may affect DWV infectivity. Studies in other RNA virus models have shown that polyphenol-rich extracts can reduce viral infectivity at the pre-entry stage by altering capsid integrity or viral adsorption [69], whereas compounds such as quercetin and luteolin have been reported to inhibit RNA-dependent RNA polymerase activity in positive-sense RNA viruses, including SARS-CoV-2 [70,71]. In the context of DWV, however, these mechanisms should be regarded as plausible hypotheses rather than demonstrated effects.

Evidence generated directly in bees nevertheless suggests that plant-derived compounds can meaningfully alter DWV-related outcomes. Boncristiani et al. [72] highlighted the capacity of natural products, including flavonoids, to modulate viral infections in pollinators through both immunomodulatory and putative antiviral effects. Pascual et al. [73] further showed that supplementation with grape pomace rich in polyphenols was associated with reduced DWV load, improved survival, and activation of immune-related genes in adult honey bees. These findings are especially relevant because they indicate that pollen-associated phytochemicals may affect DWV not only indirectly through general improvements in host condition, but also through more specific changes in infection-associated physiology.

At the same time, the biological relevance of pollen phytochemicals cannot be inferred solely from their occurrence in pollen. Their effects are shaped by compound identity, dose, post-ingestion transformation, and host physiological condition, as well as by infection status, and gut microbiota composition [74,75]. In particular, the biological activity of flavonoids and other polyphenols is strongly influenced by their molecular structure. Variations in hydroxylation patterns, glycosylation, methylation, and overall molecular conformation can affect bioavailability, antioxidant capacity, immunomodulatory properties, and potential antiviral activity [76,77]. Consequently, individual flavonoids and polyphenols may differ substantially in their biological activity. Therefore, antiviral or immunomodulatory effects observed for specific compounds cannot be assumed for all members of the same chemical class. Floral products can affect bee pathogens through both direct and host-mediated pathways, including effects on immune function and gut microbiota composition [78,79]. This complexity is reinforced by evidence showing that the phytochemical profiles of larvae and adult bees differ from those of their pollen diet, suggesting that metabolism, selective transfer, and biotransformation strongly influence actual host exposure [80]. Viewed within the resistance–tolerance framework, pollen-derived phytochemicals may influence DWV outcomes through both mechanisms. Some compounds may contribute to resistance by limiting viral replication or enhancing antiviral immune responses, whereas others may promote tolerance by reducing oxidative damage, supporting physiological homeostasis, and improving survival despite persistent infection.

**Figure 1:**
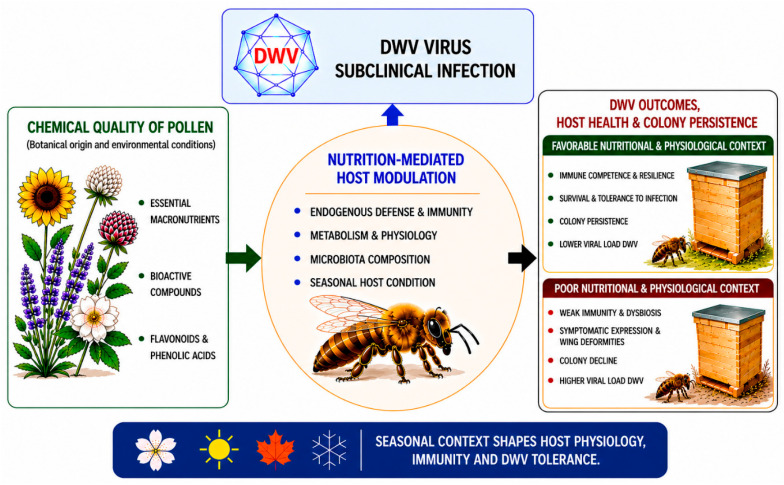
Conceptual model showing how pollen chemical quality influences honey bee immunity, DWV viral load, and colony health.

Thus, the role of pollen-derived phytochemicals should not be viewed as a uniform antiviral effect, but rather as a context-dependent modulation of infection outcomes emerging from the interaction among floral chemistry, host physiology, oxidative balance, microbiota, and environmental stress. From this perspective, the novelty of current research lies not simply in identifying phytochemicals in pollen, but in understanding how these compounds shape the physiological environment in which DWV persists, replicates, and causes damage. Pollen phytochemicals therefore emerge as candidate modulators of infection outcome rather than universally acting antiviral agents (Figure 1). This interpretation provides a more integrative framework for explaining why chemically distinct diets may lead to different patterns of viral load, survival, and tolerance in *A. mellifera* and highlights the need for bee-specific mechanistic studies that move beyond extrapolation from non-apicultural viral systems.

Direct mechanistic evidence for the effects of pollen-derived phytochemicals in the *A. mellifera*–DWV system remains limited. Therefore, much of the current functional interpretation is informed by studies conducted in other RNA virus models. Table 2 summarizes selected pollen-derived phytochemicals with reported antiviral activity in non-bee systems, highlighting their proposed mechanisms of action and their plausible relevance to DWV infection in honey bees. In this context, these mechanisms should be interpreted as working hypotheses rather than as demonstrated antiviral effects in bees.

## 7. Seasonal and Ecological Influences on Diet Quality, DWV Load, and Bee Survival

Honey bees exhibit seasonal variation in their tolerance to viral infections. The biological impact of DWV depends not only on the presence of the virus or on viral load, but also on the health status of the host and the environmental context in which the infection occurs [91]. This perspective is consistent with general frameworks in disease ecology, according to which seasonality simultaneously modulates pathogen dynamics, host susceptibility, and the environmental conditions that mediate their interaction [92]. In this sense, seasonality implies changes in the body condition of individuals and in colony organization. Differences between summer and winter bees include variations in fat body reserves, metabolism, microbiota, transcriptomic profiles, and immunocompetence [93]. In this context, tolerance to DWV should be understood as a dynamic trait, conditioned by the moment of the annual cycle in which infection occurs [94]. In support of this view, Frunze et al. [58] identified seasonal differences in the expression of immune-related genes in *A. mellifera*, reporting reduced expression of the Toll pathway ligand spätzle (spz) and the JAK/STAT pathway receptor *domeless* in winter bees compared with spring populations. The authors also proposed superoxide dismutase (SOD) and defensin-2 as potential biomarkers of colony health. These findings suggest that seasonal transitions are accompanied by shifts in immune regulation, which may influence the capacity of colonies to respond to pathogen pressure throughout the annual cycle.

This framework is especially relevant for DWV, whose epidemiology is closely linked to *V. destructor*, but is not explained exclusively by the presence of the mite. The interaction between *V. destructor* and DWV has driven the amplification, spread, and changes in virulence of this pathogen system on a global scale [15,95,96]. Longitudinal studies have shown that viral abundance, pathogen co-occurrence, and colony vulnerability change seasonally, with overwintering being a particularly critical period [97]. For example, Zhang et al. [98] reported significant seasonal variation in DWV-B prevalence and viral load, reaching 70% and 80% of colonies during May 2018 and 2019, respectively. Although the mechanisms underlying these patterns remain unclear, the authors suggested that colony health, mite levels, and the quality and abundance of food resources may contribute to seasonal viral dynamics. The potential contribution of food resource quality and abundance to seasonal viral dynamics is particularly relevant because seasonal fluctuations in pollen diversity and composition alter the nutritional environment available to colonies and may contribute to seasonal variation in resistance and tolerance to DWV infection. Temporal changes have been documented in the relative prevalence of viral variants and in their association with colony losses, especially in relation to the dynamics between DWV-A and DWV-B [99,100]. Evidence from colony-level metagenomic analyses further indicates that DWV populations can undergo seasonal genetic shifts within individual colonies, highlighting the dynamic nature of this virus beyond broader population-level patterns [101]. Given that some genotypes differ in virulence, transmission, and persistence, these transitions can modify not only the epidemiology of the virus, but also the biological consequences of infection at the colony level [102]. These virus–host–vector interactions occur within a broader ecological context in which the floral landscape and the seasonal availability of pollen can also modulate host–pathogen relationships. The composition and diversity of collected pollen change throughout the year and according to land use, affecting the nutritional basis that sustains both individual bee and the colony [103]. In intensive agricultural landscapes, colonies often face an alternation between floral abundance and scarcity, with effects on their health and their ability to cope with stressors [104]. Thus, seasonal tolerance to DWV should not be interpreted only as an intrinsic property of the host, but rather as the result of the interaction among the time of year, the context of infection, and the quality of the nutritional environment.

This way of understanding tolerance is aligned with the distinction between resistance and tolerance as alternative defense strategies against disease [31]. Applied to *A. mellifera*, this suggests that colonies may pass through periods of the year in which they do not necessarily control infection more effectively but do differ in their ability to maintain vital functions and survive in the face of viral infection. In this sense, seasonality emerges as an ecological organizer of DWV pathogenicity and raises relevant applied implications, since if tolerance to the virus changes throughout the year and depends on the ecological context of the host, then floral landscape management, resource availability during critical periods, and timely *V. destructor* control could contribute not only to limiting viral transmission, but also to reducing the probability that infection results in severe damage or colony loss [105].

## 8. Gut Microbiota as an Intermediary Between Pollen Chemical Quality and DWV Outcomes in *A. mellifera*

In *A. mellifera*, the relationship between pollen chemical quality and DWV infection should be interpreted considering the gut microbiota, which may act as an important intermediary between diet and host response [106]. The honey bee gut microbiome is relatively simple and is concentrated mainly in the hindgut, where a core set of bacterial taxa predominates, including *Lactobacillus*, *Bifidobacterium*, *Bombilactobacillus*, *Gilliamella*, and *Snodgrassella*, whereas other taxa such as *Bartonella*, *Commensalibacter,* and *Frischella* are often considered more variable or transient members [107]. This microbial community contributes to digestion, metabolism, and immune regulation, and its composition is shaped by diet, season, and environmental conditions [108]. In general, diets derived from diverse floral sources tend to support more stable and functionally diverse gut communities, whereas monofloral or artificial diets are more often associated with simplified microbiota [109]. Within this framework, pollen-derived bioactive compounds may influence host–pathogen interactions not only at the systemic level but also through changes in the intestinal environment. Experimental evidence shows that dietary phytochemicals such as caffeine, gallic acid, p-coumaric acid, and kaempferol can increase gut microbial diversity and alter the abundance of taxa such as *Snodgrassella* and *Lactobacillus*, although these effects appear to be compound-specific [110]. Similarly, Braglia et al. [111] showed that pollen from different botanical origins significantly modified the composition of the core gut microbiota and was associated with changes in immune-related hemolymph markers, highlighting the importance of pollen quality as a determinant of host physiological status. Other studies have also linked dominant symbionts, particularly *Gilliamella apicola*, to improved digestive efficiency, intestinal barrier function, and overall physiological condition [112]. Although these studies do not directly assess DWV infection, they provide a mechanistic basis for considering microbiota as a mediator of diet-dependent variation in infection outcome [113]. A complementary perspective was provided by Svobodová et al. [114], who compared *V. destructor*-surviving and *V. destructor*-susceptible honey bee populations using microbial association networks. Their results showed marked differences in microbiota assembly between the two groups and suggested that microbiome structure may contribute to resilience against viral infections, although the study was based on associative rather than mechanistic evidence. Pollen phenolics may reach the gut in partially transformed forms that selectively affect microbial composition and metabolism [115]. Similar interactions have been described in other organisms, although their specific outcome depends on compound identity, concentration, and the pre-existing microbial community [116]. In honey bees, microbial biotransformation of dietary compounds has been documented mainly in relation to nutrient metabolism and host physiology, rather than as a direct antiviral mechanism [113,117]. Consequently, microbiota-mediated effects on host physiology may help explain why bees fed chemically richer diets often show lower viral loads and improved survival. This interpretation is consistent with studies showing that disruption or absence of the gut microbiota is associated with broad changes in host gene expression affecting immunity, metabolism, development, and behavior [118]. Nevertheless, in the honey bee–DWV system, current evidence does not support a direct causal role of the microbiota in controlling viral replication. Rather, the gut microbiome should be viewed as part of a broader diet-dependent physiological network through which pollen chemical quality may shape tolerance to infection, survival, and colony health in honey bees.

## 9. Limitations and Gaps in Knowledge

Despite increasing evidence that pollen chemical quality can influence DWV infection outcomes in honey bees, important methodological and conceptual limitations still prevent the development of robust and predictive models. One of the main challenges is the high phytochemical variability of pollen, which arises from taxonomic, environmental, and seasonal differences [119]. This variability complicates comparisons among studies and limits reproducibility. The concentration of flavonoids and other bioactive metabolites can vary considerably according to the botanical and geographical origin of bee pollen, making it challenging to directly compare biological effects among studies [120,121].

A second major limitation lies in the heterogeneity of experimental designs used to evaluate the effects of pollen and bioactive compounds on DWV. Studies differ in viral inoculation procedures, developmental stages examined, laboratory versus field settings, control diets, and concentrations of phenolic compounds, often preventing direct comparison of results [73,122]. In addition, many experiments rely on complex plant extracts or pollen mixtures whose chemical composition is only partially characterized, which limits the identification of the metabolites responsible for the observed responses [49].

Knowledge of the bioavailability and metabolism of flavonoids and phenolic acids in bees also remains surprisingly limited. It is still largely unknown how these compounds are absorbed, biotransformed, distributed, or stored, and which concentrations they reach in physiologically relevant tissues such as the hypopharyngeal glands, brain, or gut [80]. This uncertainty makes it difficult to connect dietary composition with actual host exposure and biological effect. The interaction between phenolic compounds and the gut microbiota adds further complexity, since microbial biotransformation may generate secondary metabolites capable of influencing host physiology and immunity, yet the underlying mechanisms remain poorly resolved [106]. The lack of integrated metabolomic approaches therefore remains a key obstacle to establishing causal links among pollen chemistry, microbial activity, and DWV modulation.

Another unresolved issue concerns the dose-dependent effects of polyphenols. Although these compounds are often discussed in relation to antioxidant and protective functions, some studies indicate that high concentrations may act as pro-oxidants and induce oxidative or cytotoxic effects [123]. This pattern is consistent with hormetic responses, in which the effects of bioactive compounds vary according to dose and biological context [124]. This suggests that the biological activity of pollen phytochemicals cannot be interpreted independently of dose, diet matrix, or physiological context. In parallel, intercolony variability in responses to supplemental diets indicates that genetic, and possibly epigenetic, factors may also influence antiviral responses, although these sources of variation are rarely incorporated into the same experimental framework [4].

A further gap concerns the translation of laboratory findings to field conditions. Many studies report beneficial or antiviral-associated effects of supplemental diets under controlled conditions, but their expression in real colonies is shaped by seasonality, floral variability, *V. destructor* mite level, pathogen co-occurrence, and environmental stress [125]. As a result, effects detected in caged-bee assays cannot be directly extrapolated to colony-level resilience in complex landscapes.

Overall, these limitations indicate that the relationship between pollen chemical quality and DWV cannot yet be understood through single-factor explanations. Progress in this field will require more standardized experimental designs, chemically well-characterized diets, integrated metabolomic and microbiome analyses, and field-scale studies capable of capturing seasonal and ecological variation. Addressing these gaps will be essential not only to clarify how pollen chemistry shapes DWV outcomes, but also to support the development of evidence-based nutritional strategies to improve colony resilience under increasingly challenging environmental conditions.

## 10. Conclusions

The available evidence indicates that the outcome of deformed wing virus (DWV) infection in *A. mellifera* is shaped not only by viral pressure, but also by the nutritional and physiological context in which infection occurs. Within this framework, pollen chemical quality emerges as an important determinant of host condition, influencing immune function, oxidative balance, and the capacity of bees to tolerate infection. Chemically rich and diverse pollen diets are more consistently associated with improved survival and physiological performance than with uniform reductions in viral load, suggesting that their main contribution may lie in enhancing host resilience rather than in acting as direct antiviral factors. Together, these findings support the view that pollen quality influences DWV outcomes through a combination of resistance- and tolerance-related mechanisms, with the latter appearing more consistently associated with improved survival and colony performance. This interpretation also highlights the potential role of the gut microbiota as an intermediary through which pollen-derived compounds may influence host physiology and infection outcomes, although the specific mechanisms involved remain insufficiently resolved.

At the same time, current knowledge is constrained by the high phytochemical variability among pollen sources, the heterogeneity of experimental designs, the limited understanding of compound bioavailability and metabolism in bees, and the difficulty of extrapolating laboratory findings to field conditions. Future research should therefore combine chemically characterized diets, bee-specific mechanistic studies, microbiota and metabolomic approaches, and field-scale experiments that incorporate seasonal and ecological variation. Such advances will be essential to clarify how pollen chemical quality shapes DWV outcomes and to support the development of evidence-based nutritional strategies aimed at strengthening colony resilience under increasingly challenging environmental conditions.

## Figures and Tables

**Table 1:** Effects of pollen quality and pollen-based diets on DWV infection outcomes and related health traits in honey bees.

Study Focus	Diet or Pollen Treatment	Experimental System	Response Variables	Pathogen(s) Evaluated	Main Findings	Reference
Diet type and DWV load	Natural pollen, protein supplements, or sucrose syrup	Individual bees (laboratory)	Protein levels, hypopharyngeal gland acini size, viral titer	DWV	DWV titers were lowest in pollen-fed bees and highest in sucrose-fed bees; viral load declined over time in bees fed pollen.	[55]
Pollen supplementation and colony-level viral load	Colonies in an *Eucalyptus grandis* plantation with or without polyfloral pollen patty supplementation	Colonies (field)	Colony strength, *Nosema* infection level, brood, RNA virus load	RNA viruses, *Nosema* spp.	Supplemented colonies showed higher DWV and ABPV titers but were stronger; viral titers remained below threshold for colony decline	[32]
Effect of pollen nutrition on gene expression in healthy and *Varroa*-parasitized honey bees (nutrigenomics)	Pollen + sugar syrup vs. sugar syrup only	Laboratory conditions	Transcriptome (digital gene expression/DGE-tag profiling); nutrient-sensing pathways, metabolic genes, longevity genes, antimicrobial peptide genes	*V. destructor* associated viral populations	Pollen activated nutrient-sensing, metabolic and immune pathways; however, the virus-promoting effects associated with *Varroa* parasitism persisted despite pollen consumption.	[29]
Contrasting pollen-based diets and viral load	Pollen-based diets from different floral sources	Individual bees (laboratory)	Immune gene expression, survival, viral load	DWV-A	Pollen diets reduced the viral load of DWV-A from 10^13^ to 10^5^–10^6^ copies/bee and increased survival rates to 91%. In addition, they modulated the expression of immune genes.	[52]
Pollen, *Varroa*, and DWV via behavioral maturation genes	Pollen-*supplemented* vs. pollen-free diet in *Varroa*-infested bees	Individual bees (laboratory)	Lifespan, Vitellogenin, Juvenile Hormone Esterase, immune-related gene expression, DWV load	DWV (*Varroa*-associated)	Pollen increased the lifespan of mite-infested bees by reversing premature behavioral maturation induced by *Varroa* and was associated with higher AMP expressions and lower DWV load.	[56]
Field pollen supplementation and colony demography	Supplemental pollen feeding	Colonies (field)	Colony population, brood, survival; DWV and *Varroa* levels	*V. destructor* and DWV	Pollen-fed colonies were larger, had more brood, and survived longer, whereas DWV and *Varroa* levels were similar between fed and unfed colonies. The survival benefit was therefore associated with improved colony growth rather than with a consistent reduction in pathogen or mite levels.	[53]
Diet protein composition and viral outcomes	Artificial diets: free amino acids vs. intact proteins	Caged bees (laboratory)	Vitellogenin, MRJP1, survival	DWV	Improved nutritional biomarkers (*vg* and *mrjp1* expression), with contrasting effects on DWV dynamics depending on whether nutrients were supplied as intact proteins or free amino acids.	[57]
Artificial diets and DWV expression	Artificial diets varying in protein composition and digestibility	Individual bees (laboratory)	Protein metabolism, molecular health markers, DWV relative expression	DWV	Diet composition was associated with differences in protein digestibility, host molecular markers, and DWV relative expression	[58]

Note: Comparisons across studies should be interpreted with caution because of differences in experimental systems, viral strains, life stages, and response variables evaluated.

**Table 2:** Pollen-derived phytochemicals with antiviral activity reported in non-bee systems: potential mechanistic hypotheses for future studies of DWV in honey bees.

Compound	Chemical Classification	Viral Models Studied	Reported Antiviral Mechanism	Affected Stage of Viral Cycle	Relevance to DWV in Honey Bees	References
Quercetin	Flavonol	SARS-CoV-2, rhinovirus, influenza, and other RNA viruses	RdRp inhibition; interference with endocytosis pathways; redox and immune modulation.	Viral entry, genome replication, translation	Plausible candidate for modulation of viral replication and host oxidative balance in DWV-infected bees	[71]
Chlorogenic acid	Phenolic acid	Influenza A (H1N1/H3N2) and other viruses	Neuraminidase inhibition; reduced viral replication; antioxidant and anti-inflammatory activity.	Viral release/spread, replication	Potential modulator of oxidative stress and infection-associated physiological damage rather than a demonstrated direct antiviral in bees	[81]
Luteolin	Flavone	Coronavirus, influenza, enterovirus, RSV; SARS-CoV-2	Inhibition of viral entry and replication; modulation of the host response.	Viral entry, genome replication	Plausible candidate for interference with viral replication and for immunomodulatory effects in the honey bee–DWV system	[70,82]
Kaempferol	Flavonol	Influenza, coronavirus, hepatitis B, enterovirus	Inhibition of viral entry or fusion and replication; modulation of host pathways.	viral entry, replication	Potential contributor to host resilience and antiviral defense, although evidence in bees remains indirect	[83]
Apigenin	Flavone	Enterovirus 71	Inhibition of viral IRES activity; modulation of the JNK pathway; suppression of replication.	Genome replication, viral translation	Plausible mechanistic candidate for modulation of viral translation-related processes, pending bee-specific evidence	[84]
Myricetin	Flavonol	SARS-CoV	Inhibition of the viral helicase nsP13 and other viral enzymatic activities	Genome replication, viral protein processing	Hypothetically relevant to DWV replication through interference with viral enzymatic functions, but not demonstrated in bees	[85]
Pinocembrin	Flavanone	Zika virus and other RNA viruses	Inhibition of post-entry processes; reduction in viral RNA and viral protein synthesis	Post-entry replication	Plausible candidate for limiting intracellular viral replication and associated cellular stress in bees	[86]
Caffeic acid	Hydroxycinnamic acid	HCV, HBV, HSV-1	Antioxidant activity and inhibitory effects on viral replication	Viral replication, host antioxidant response	More likely relevant as a modulator of oxidative balance and host condition during DWV infection than as a confirmed direct antiviral	[87]
Ferulic acid	Hydroxycinnamic acid	Influenza, VSR	Antioxidant activity and modulation of NF-κB/host inflammatory response	The host’s response to oxidative stress and inflammation	Plausibly relevant to tolerance mechanisms by limiting infection-associated oxidative and inflammatory damage	[88]
Rutin	Flavonol	EqHV-8, SARS-CoV-2	Reduction in viral infection and oxidative stress; activation of the Nrf2/HO-1 pathway	Viral replication, antioxidant response	Potential candidate for improving tolerance to DWV through antioxidant and cytoprotective effects	[89]
Naringenin	Flavanone	SARS-CoV-2, Zika, dengue and other RNA viruses	Putative inhibition of 3CLpro; reduced viral internalization/entry; decreased viral titter in flaviviruses; anti-inflammatory modulation	Viral entry, post-entry replication	Plausible multifunctional candidate linking antiviral and host-protective effects, although evidence for DWV remains indirect	[90]

Note: Mechanisms described for mammalian RNA viruses are presented as plausible working hypotheses, not as demonstrated effects in the *A. mellifera*–DWV system.

## Data Availability

No new data were created.
